# Patterns of use, perceptions, and cardiopulmonary health risks of cigar products: a systematic review

**DOI:** 10.1186/s12889-023-17216-z

**Published:** 2023-11-28

**Authors:** Comreen Vargees, Andrea M. Stroup, Taylor Niznik, Delaney Dunn, Riley Wyatt, Cosima Hoetger, Ziyad Ben Taleb, Amy M. Cohn, Caroline O. Cobb, Jessica L. Fetterman

**Affiliations:** 1 Evans Department of Medicine and Whitaker Cardiovascular Institute, Boston University School of Medicine, 600 Albany Street, Boston, MA USA; 2 Westat, 1600 Research Boulevard, Rockville, MD USA; 3 TSET Health Promotion Research Center, Stephenson Cancer Center, University of Oklahoma Health Sciences Center, Oklahoma City, USA; 4 Department of Psychology, Virginia Commonwealth University, 806 W Franklin St, Richmond, VA 23284 USA; 5 Center for the Study of Tobacco Products, Virginia Commonwealth University, 100 W Franklin St, Richmond, VA 23220 USA; 6 Institute for Integrative Health Care and Health Promotion (IGVF), Witten/Herdecke University, Alfred-Herrhausen-Straße 50, Witten, 58455 Germany; 7 Department of Kinesiology, College of Nursing and Health Innovation, University of Texas at Arlington, Arlington, TX USA; 8 Department of Pediatrics, College of Medicine, University of Oklahoma Health Sciences Center, Oklahoma City, USA

**Keywords:** Cigar, Little cigar, Cigarillo, Blunt, LCC, Cardiovascular, Pulmonary, Cardiopulmonary

## Abstract

**Objective:**

A systematic review was conducted to evaluate the use patterns, health perceptions, and cardiopulmonary health effects of cigars.

**Data sources:**

PubMed and Google Scholar were searched for peer-reviewed articles published between June 2014 and February 2021. Search keywords included cigars, cigarillos, little cigars, and cardiopulmonary health outcomes.

**Study selection:**

Of 782 papers identified, we excluded non-English articles, review articles, commentaries, and those without empirical data on cigars. Three coders independently reviewed all articles and compared codes to resolve discrepancies. 93 articles met the inclusion criteria and were included.

**Data synthesis:**

Cigars have evolved from premium cigars to encompass little cigars and cigarillos (LCCs). LCCs are available in an array of flavors and at a price advantage, and as a result, are used by different groups compared to premium cigars. LCCs are more frequently used by youth, young adults, and those who identify as Black/African American. LCCs are often used in combination with other tobacco products, alcohol, and cannabis. Despite limited regulation, cigars generate smoke of a similar composition as cigarettes. Among the studies identified, evidence suggests that cigar use is associated with cardiovascular and pulmonary toxicity. Higher all-cause and cancer-related mortalities are associated with cigar use, particularly with more frequent and deeper inhalation, compared to non-tobacco users.

**Conclusions:**

LCCs are used more frequently by at-risk groups compared to premium cigars. Recent studies evaluating cigar cardiopulmonary health effects are limited but suggest cigars have similar health risks as conferred by cigarette smoking. With the use of LCCs and targeted marketing on the rise among high-risk groups, there is a critical need for continued research in this area.

## Introduction

While cigarette consumption has steadily decreased in recent years, cigar use is on the rise, especially among groups at higher risk, including youth, young adults, those who have been socially marginalized, and those who identify as Black/African American. The latter two groups are of particular concern considering their higher prevalence of cancer, health-related morbidities, and worse tobacco-related health outcomes [1–3]. Among United States (U.S.) adults (ages 18+), cigars are the third most commonly reported tobacco product, after cigarettes and electronic cigarettes (e-cigarettes) [4]. Cigar use is highest among U.S. adults (ages 25–44), with 5% reporting current use (past 30-day) of any cigar type in the 2021 National Health Interview Survey dataset [4].

Cigars are classified into three categories: (1) large/premium cigars, (2) cigarillos, and (3) little cigars (Table 1) [5]. All cigars are defined as a roll of tobacco filling wrapped in a substance at least partly consisting of tobacco leaf [6]. Cigarillos more closely parallel premium cigars in size, shape, use behavior, and smoking topography [6]. Cigarillos are predominantly unfiltered, although some have plastic/wooden tips, and are often flavored. Cigarillos are used to create blunts, involving the removal of some or all of the tobacco filler and replacement with cannabis. Unlike cigarettes and little cigars, a single cigarillo is less likely to be finished in one session [7, 8]. Little cigars, often coined “brown cigarettes” or “filtered cigars,” closely resemble cigarettes in size, feel, and tobacco content, and are typically sold in packs of 20 [6].

**Table 1:** Types of Cigar Products

Cigar Type	Alternative Names	Classification	Available in Flavors	Package Sizes	Modifications
Premium Cigars	Large cigars	Weigh more than 3 lbs/1000 units and do not contain a filter.		≥ 1	
Cigarillos	Large cigars, Flavored cigars	Weigh more than 3 lbs/1000 units but are often longer and slimmer than premium cigars. Do not contain a filter. May have a plastic/wood tip.	√	Packs range from 1–5	Blunts (removal of tobacco and replacement with cannabis) Hyping/freaking (removal of the inner layer of filter paper)
Little Cigars	Small Cigars, Filtered Cigars, Brown cigarettes	Similar size and shape as cigarettes and like cigarettes, often contain a filter. Like all cigar products, contains aged and fermented tobacco, and a wrapper containing, in part, tobacco leaf.	√	Packs of 20	

Cigars generate smoke of a similar composition as cigarettes and in the case of a few toxicants, greater levels [9, 10]. Cigar smoking is associated with a number of health outcomes including coronary heart disease [11] and chronic obstructive pulmonary disease (COPD); and confers similar cancer-related risks when compared to smoking cigarettes [12, 13]. Dual and poly-tobacco use of cigars with other tobacco products likely increases exposure to harmful carcinogens and toxicants beyond the use of either product alone, which may exacerbate the health risks associated with combustible tobacco product use [13, 14]. Evaluation of the current state of knowledge regarding the association of the use of new and emerging cigar products and contemporary cigar use patterns with cardiovascular and pulmonary toxicity and outcomes is needed to inform cigar product regulations.

The increasing popularity of cigars in the U.S. is driven, in part, by the absence of regulation, a price/tax advantage compared to cigarettes, and the use of characterizing flavors in cigar products not present in cigarettes [15]. Some groups misperceive cigars and modified cigar products like blunts as less harmful than cigarettes [16–19]. However, strides have been made to tighten cigar product regulation in response to increased use. In 2016, the U.S. Food and Drug Administration (FDA)’s regulatory authority of tobacco products was extended to include cigars [20]. In November 2018, the FDA announced intentions to “propose a policy…to ban flavors in cigars” [21]. While no such ban has yet been enacted, in April 2021 the FDA announced its commitment to propose new product standards that would ban menthol and other flavors from cigars in an effort to reduce youth initiation and health disparities, while improving tobacco cessation efforts [22]. A secondary outcome of a flavor ban may be a decrease in cigar-related morbidity arising from a hypothetical decline in cigar use.

In light of the increasing prevalence of cigar smoking in the U.S. and the FDA’s new regulatory authority over cigars, studies are needed to evaluate the cardiopulmonary health effects associated with contemporary cigar use patterns. Information regarding the health effects of contemporary cigar smoking patterns may help inform new regulatory efforts to protect public health, especially among groups disproportionally affected. A systematic review encompassing relevant literature up to 2014 found very few studies (< 20) that assessed the cardiopulmonary health effects of cigars [13]. With the FDA’s intentions to regulate flavoring in cigars in mind, we sought to provide an update on the use patterns, perceptions of risk, and the cardiopulmonary health effects and outcomes associated with cigar use. Due to differences in the use patterns, whenever possible, we delineate between the different cigar product types. Given the high prevalence of flavored cigar product use, where applicable, we address the health effects associated specifically with the use of flavored cigars.

## Methods

**Fig. 1:**
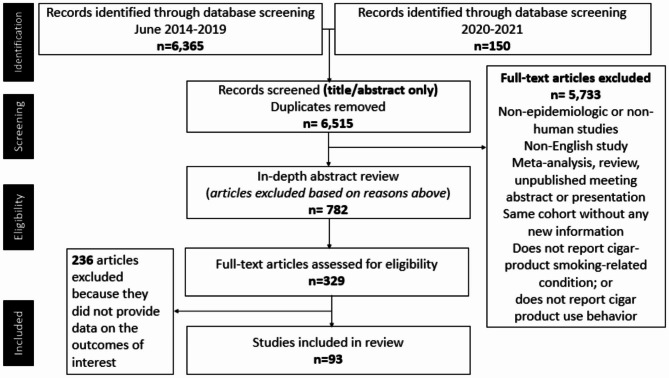
Flowchart of article selection

PubMed and Google Scholar were searched for articles published between June 2014 (year a similar systematic review was published [13]) and June 2019 with 6,363 articles identified. The search was updated in 2021 to include publications from July 2019 through February 2021, yielding an additional 150 eligible articles. Both databases were searched using consistent combinations of the following search terms: cigar(s), cigarillo(s), and little cigar(s). The specific term “filtered cigar(s)” was not included in the literature search as no additional articles were retrieved when included with the other terms. After screening paper titles and removing duplicates, a total of 782 article abstracts were examined to ensure that each met the predefined inclusion criteria (Table 2). 329 full-text articles were coded by the co-authors and data extraction was performed using a template designed by the primary authors. Each article was coded twice, by two separate, independent coders to ensure consistency in data extrapolation and synthesis. Coding discrepancies were reviewed by a third coder and resolved by the primary authors. Any article that did not meet eligibility criteria and/or did not provide data on the outcomes of interest was excluded prior to writing the paper. We did not exclude publications based on country of origin. In total, 93 articles were included in the paper (Fig. 1; Table 3). We followed the recommendations outlined in the Preferred Reporting Items for Systematic reviews and Meta-Analyses guidelines for systematic reviews [23, 24].

**Table 2:** Article Inclusion and Exclusion Criteria

***Inclusion Criteria***	
Peer-reviewed	
Contain empirical data on traditional cigars, little cigars, cigarillos, or other cigar products	
Contain data addressing topics of interest:	
	Toxicity
	Flavoring
	Perceptions of harm
	Addiction
	Cardiovascular health outcomes (atherosclerosis, coronary heart disease, stroke, peripheral artery disease, cardiovascular disease, blood pressure, heart rate, narrowing/damage of blood vessels, damaged heart/vascular tissue, heart attack/failure, chest pain, arrhythmia, blood clot, cardiovascular, heart, mortality, death)
	Pulmonary health outcomes (asthma, bronchitis, chronic obstructive pulmonary disease, lung cancer, pneumonia, lung infection, pulmonary edema/embolus, lung function, pulmonary, lung, mortality, death)
***Exclusion Criteria***	
Review articles	
Commentaries	
Unpublished academic papers	
Meeting presentations	
Tobacco industry funded research	

**Table 3:** Types of Studies Identified

***Cigar Use Patterns and the Role of Flavored Cigars***
Single, dual, poly-use with other cigar products or with other tobacco products [16, 25–27, 31–33, 47, 48]
Role of flavors and flavor type in cigar product use [28, 34, 36, 38–41, 43, 46, 89, 100, 103, 104]
Association of nicotine or indices of addiction with cigar product use [6, 33, 55–60, 62]
Variations in patterns/correlates of use, or tobacco use outcomes across cigar product type (large cigars, cigarillos, filtered cigars) [6, 7, 26, 31–33, 37, 44, 45, 47–49, 51, 56, 105–107]
Association of flavored cigar products with use patterns: A) Types of flavored cigars used among young adults [34, 35, 89] B) Characteristics of flavors in markets where marijuana is legal [43] C) Association of flavoring with prevalence and patterns of use among youth, young adults, and adults in the US and Canada [36, 38–44, 46, 104]
***Use of multiple substances among users of cigars***
Prevalence, reported patterns of use, and correlates of single, dual, and poly-use tobacco with other substances among U.S. young adults [47, 48]
Youth cigar use [16, 48, 49]
***Smoking topography of cigars and biomarkers of exposure***
Smoking topography, biomarkers of exposure, mainstream smoke composition [6, 7, 9, 50–54, 56, 59, 108]
Biomarkers of exposure associated with cigar use or following acute use of cigar product or cigarette [50–52, 55, 109]
Mainstream smoke composition of LCCs [6, 7, 9, 53, 54, 59, 108]
***Cigar nicotine content, delivery, dependence***
Laboratory smoking studies examining nicotine biomarkers and measures of dependence [6, 7, 51, 58, 59]
Studies analyzing nicotine content in different cigar products [55–57]
Studies evaluating the role of cannabis in concurrent and subsequent cigar use [33, 61, 62]
***Perceptions of harm of cigar smoking***
Perceived harmfulness of cigar products [17–19, 26, 63–86]
***Cardiopulmonary health effects of cigars***
Evaluation of the relation between short and long-term cardiopulmonary health measures and outcomes associated with cigar use behaviors and smoking topography [6, 58, 61, 91–98, 103]
Harm of less frequent cigar smoking that indicates daily cigar smokers are at higher risk for cardiovascular and pulmonary disease [91–93, 98]
Similar nicotine exposures between cigar products and cigarettes indicate changes in HR, vascular function, and spirometry, suggestive of cardiopulmonary toxicity associated with cigar use [6, 52, 58, 61, 95, 96, 103]
Toxicity of cigar smoke exposure or flavoring additives on bronchial or vascular epithelial cells in vitro [100, 107]

## Results

### Cigar use patterns and the role of flavored cigars

The prevalence of cigar use among adults was 3.5% in a 2021 nationally representative survey [4]. Among adults reporting cigar use in the past 30 days, nearly half were between the ages of 18–44 years, consistent with the higher prevalence of cigar use among young adults found in other studies [16, 25]. Among adults who smoke, individuals who use LCCs are more likely to be male, have a lower income, report having tried menthol tobacco products, and report lifetime substance use disorder symptoms [3, 26]. Non-Hispanic, Black individuals were more likely to report daily and established cigar smoking, especially those reporting cigarillo use, compared to White, non-Hispanics [3]. Using a latent class analysis to classify adult cigarette smokers based upon demographic characteristics, tobacco and substance use, desire to quit smoking, and a measure of mental health - three classes of smokers emerged. Of interest, one class consisted predominantly of Black males who reported current dual use of LCCs and menthol cigarettes [27].

#### Cigar use among youth and young adults

Many studies evaluated cigar use patterns in youth and young adults by cigar product type. In this review, we considered blunts as a separate and unique cigar product. In nationally representative samples in the U.S., LCCs and blunts were more popular than large cigars among Black individuals [16, 25, 27–30] and young adults [16, 25]. Among young adults (18–34 years of age), past 30-day onset of large cigar use was more likely among participants who reported past 30-day use of LCC, pipe tobacco, and chewing tobacco [31]. Past 30-day onset of LCC use was more likely for participants who reported past 30-day cigarette, large cigar, and hookah use. The age of initiation differed across cigar product types with LCCs associated with an earlier age of initiation (< 17 years) versus premium cigars; and LCC use was positively correlated with current and future cigar use behavior and patterns [16, 25, 28, 31].

Among 9th -grade students, youth of parents with lower reported incomes were more likely to be current tobacco users, including cigar users; however, specific tobacco use rates were not reported [32]. Another study of 9th -grade students found that ever use of combustible cigarettes at baseline was associated with higher odds of reporting current cigar (past 30-day) use at follow-up [33]. A qualitative study examined changes in cigar use patterns, harm perceptions, access, and motivation to quit before and after the COVID-19 outbreak, by cigar product type. Participants reported an increased quantity and frequency of cigarillo and blunt use, but a decreased quantity or frequency of large cigar use, after the pandemic [29].

#### Use of flavored cigars among youth and young adults

Flavored cigar products are particularly popular among youth and young adults [34, 35]. Initiation of cigar use with a mint/menthol flavored cigar was associated with continued cigar use among youth and young adults when compared with cigar use initiation with non-flavored cigars [36]. In 2019, of the youth who reported current use (past 30-day) of cigars, 41.9% reported the use of a flavored cigar product, consistent with earlier studies [37–39]. Among youth who reported using ≥ 2 tobacco products, the most commonly used combination was e-cigarettes and cigars (17.2%) [37]. Although cigar products were grouped together, the higher prevalence of cigarillo use among youth would suggest that the flavored tobacco products (e-cigarettes and cigarillos) may be contributing to youth and young adult tobacco use.

Five studies found that the most popular flavors of cigars were fruit flavors [34, 40–42], particularly in areas where cannabis was legal recreationally [43]. Flavored cigar use was associated with being younger and having higher socioeconomic status [44]. Additionally, flavored LCC packaging with colorful images depicting alluring flavor profiles are implicated in increasing the appeal of LCCs [40, 45]. Availability of flavored LCCs was associated with increased odds of LCC initiation and susceptibility to cigar use among youth and young adults [40, 46].

### Individuals who use cigars often also use other multiple substances

Using the Population Assessment of Tobacco and Health (PATH) Study (2014–2015), the prevalence and correlates of tobacco and cannabis use were assessed in a national sample of U.S. young adults (18–24 years) [25]. Of the young adults reporting co-use of tobacco and cannabis, 43.4% reported using cigarillos compared to 20% who reported use of premium cigars. Polytobacco use (3 + products) and higher alcohol use was associated with the use of cannabis in the presence/absence of tobacco. The top 10 most commonly reported patterns of single, dual, and polytobacco use with other substance use among young and older adults (25 + years) were also identified [47]. Among young adults, co-use of cigars with alcohol, cigarettes, or cannabis emerged in the 9th and 10th most commonly reported patterns, endorsed by 1.2% of young adults. Thus, when examined in reference to other substances of abuse like alcohol and cannabis, cigars appeared to be less popular compared to the use of cigarettes, alcohol, or cannabis [47]. A pattern of exclusive cigar use did not emerge, further highlighting that individuals who use cigars typically use cigars with other products and substances.

Among high school samples, past 30-day poly-tobacco use and use of large cigars and cigarillos was higher among those who use both cannabis and tobacco compared to individuals who only use tobacco [48]. Evaluation of demographic and psychosocial correlates across various combinations of cigar, blunt, and non-blunt cannabis use patterns revealed that past 30-day use of non-cigar tobacco products was most prevalent among individuals who use both cigars and blunts [16]. Black youth were more likely to initiate tobacco use with a cigar product; furthermore, Black youth who used a cigar product first were more likely to transition to cigarette use [16, 49]. Thus, youth who use cigars were more likely to use other tobacco products and cannabis [16, 49]. The compendium of literature suggests that cigar use, particularly LCC use, is associated with poly-tobacco and co-substance use in both young adults and adults. Moreover, we found that flavors contribute to the use and popularity of cigar products.

### Smoking topography of cigars and biomarkers of exposure

Smoking topography determines the exposure to toxicants, which has disease implications. Individuals who use non-cigarette tobacco products (including individuals who use LCC) had lower urinary nicotine, cotinine, total nicotine equivalents, and 4-(methylnitrosamino)-1-(3-pyridyl)-1-butanol (NNAL) levels compared to individuals who smoke only cigarettes, despite reporting similar past 7-day tobacco product use [50]. The differences in smoking topography between the products could account for the differences in exposure, but smoking topography was not evaluated in this study.

In contrast, interventional lab studies have shown that cigar use results in similar, sometimes higher, exposure to toxicants compared to cigarettes. In a randomized crossover study of individuals who use both little cigars and cigarettes, smoking a little cigar resulted in greater CO exposure compared to a cigarette [51]. Dual users of cigarettes and cigars who smoked a Phillies Blunt large cigar had similar nicotine exposures as to when they smoked their own brand of cigarette, but greater CO exposure when the quantity of product smoked was taken into account [52]. Another lab study showed that individuals who use both cigarettes and cigarillos smoked both products similarly, with the exception of the puff topography variables related to the size of the products [53]. Despite differences in puff topography, dual users achieved similar circulating nicotine levels when smoking either a cigarillo or cigarette, suggestive of self-titration [53]. After adjusting for the amount of product smoked or total puff volume, cigarillo smoking resulted in greater exhaled CO compared to cigarette smoking [53].

The mainstream smoke composition of various cigar types has been evaluated with a smoking machine simulated to elicit topography profiles of individuals who smoke cigars. The mainstream smoke generated by cigarillos contained higher levels of volatile organic compounds compared to little cigars or cigarettes, even after adjusting for nicotine content [9, 53]. However, on a per-gram basis, little cigars generated mainstream smoke that contained greater levels of nitrosamines, acetonitrile, and acrylonitrile compared to cigarillos [9]. Similarly, on a per-puff basis, five carbonyls were found to be higher in little cigar-generated mainstream smoke compared to filtered cigars or cigarettes [54].

### Cigar nicotine content, delivery, and dependence

#### Cigar nicotine content and exposure

Three studies conducted compositional analyses of cigar smoke to determine the nicotine content and yields. Although the tobacco pH was similar across the cigar products, physical characteristics and nicotine content varied across 20 popular large cigars and cigarillos, with significant intra-brand variation [55]. Nicotine levels in little cigars were either similar to or surpassed levels found in cigarettes [56]. The wrappers of large cigars and cigarillos were shown to contain measurable quantities of nicotine [57]. Nicotine exposure was similar when a cigarillo remained unmodified or was “hyped,” which involves the removal of the inner tobacco liner with the intent to reduce the harmful effects [58]. While cigarette smoking delivered more nicotine per gram of tobacco consumed, individuals who use both cigarettes and cigars modified their cigar smoking topography by taking more puffs at a greater velocity and utilizing more time to smoke the cigar, resulting in self-titration [52].

#### Subjective responses to cigar use

The subjective responses differed across cigar products in comparison to cigarettes [51, 58, 59]. Regardless of the cigar product type, cigar smoking was found to reduce cravings and urges to smoke [59]. No differences were found across cigar product types regarding the positive sensory aspects of smoking (reward, sensation, satisfaction). In contrast, another study showed that the subjective responses to smoking, specifically reward, satisfaction, and craving reduction, were greater following cigarette smoking versus little cigar smoking [51]. Evaluation of the subjective responses to smoking a Black & Mild cigarillo three different ways (unmodified; “hyped”; or “sham” (unlit product), revealed that the “hyped” cigarillo was rated as more satisfying, pleasant, and appetizing (e.g., “tasted good”) compared to the unmodified cigarillo and sham smoking [58].

#### Dependence associated with co-use of cigars and cannabis

Three studies assessed the relation of cannabis use in cigars with measures of dependence. Blunt smoking has been hypothesized to increase the perceived “high” of smoking either product alone. In one study [60], lifetime or past month use of both cannabis and cigars was associated with subsequent current cigar use and lower rates of tobacco quit attempts, indicative of increased nicotine dependence [61]. Both blunt and non-blunt cannabis use was associated with combustible tobacco product use at follow-up, suggestive of increased risk of nicotine dependence; although this study did not directly examine nicotine dependence [33, 62]. Hence, cigar and blunt use are associated with nicotine dependence and sustained tobacco use.

### Perceptions of harm of cigar smoking

#### Perceptions of cigar smoking harm among adults

Individuals who smoke and those who have never used tobacco have varying perceptions of the safety of cigar products [17–19, 26, 63–87]. Adults were found to believe that cigarettes are the most harmful tobacco product followed by cigarillos, filtered cigars, smokeless tobacco, pipes, premium cigars, dissolvable tobacco, hookah, and e-cigarettes [73]. Similarly, PATH Study data from 2014 revealed that the majority of adults perceived smoking any type of cigar to be equally as harmful as smoking cigarettes [77]. Across all ethnicities evaluated, non-Hispanic Black individuals who currently smoke have the highest cigar harm perceptions [76]. Four studies found comparable results, with over half of adults indicating that LCCs are highly addictive and pose similar harm as smoking cigarettes [19, 26, 86, 87]. Collectively, among adults, cigar products are perceived to have similar harm to cigarettes.

#### Perceptions of cigar smoking harm among young adults

Several studies found less perceived harm towards cigar smoking among young adults. A national survey, comprised of 2871 young adults (aged 18–34 years), indicated that 14% of respondents believed that cigars were less harmful than cigarettes [65]. Among college students, nearly half thought that cigarillos and cigars were less harmful than cigarettes [63, 64], and individuals who use tobacco perceived cigars as less harmful compared to individuals who do not use tobacco [66]. In focus groups of 43 Black college students, the majority of students held positive views of LCCs and believed that LCCs were healthier and less addictive alternatives to cigarettes [67]. Therefore, college-aged students appear to perceive cigar products as less harmful than cigarettes.

#### Perceptions of cigar smoking harm among youth and other vulnerable groups

The perceived safety of cigar use varies across youth and groups that have been socially marginalized. In two analyses of the PATH Study, 57% [79] and 60.2% [80] of youth believed that cigars/cigarillos/filtered cigars cause a great deal of harm. Approximately 30.6% of youth considered cigars more harmful than cigarettes, followed by smokeless tobacco, pipes, and hookah [80]. Interestingly among youth, a greater number of nonsmokers, compared to current smokers, agree that cigarillos are less risky than cigarettes [69]. Individuals aged 14–17, compared to those aged 12–13, perceived all tobacco products, except for smokeless tobacco, as less harmful than cigarettes [71]. Black individuals who smoke cigarettes were more likely to perceive flavored LCCs as less harmful and easier to quit than cigarettes [19]. Two other studies revealed that individuals with lower educational attainment and those working in healthcare believe that cigars and cigarillos were safer than cigarettes [68, 84]. However, the perceived harmfulness of cigar products has increased among youth over time [70].

In a study using 2016 data from the Truth Initiative Young Adult Cohort Study, 33 (11.6%) sexual and gender minorities (SGM) and 322 (11.1%) non-SGM believed LCCs were less harmful than cigarettes [78]. A cross-sectional study that surveyed individuals experiencing homelessness who use tobacco (N = 470) found that more than two-thirds of participants viewed cigar consumption as harmful to health [75]. In contrast, focus groups of unaccompanied young adults experiencing homelessness revealed that these individuals did not associate LCCs with negative characteristics, and the associated health effects were hardly mentioned [72]. One possible reason for the lack of perceived harm is that youth endorse positive affect to LCCs, thus influencing their risk perception [18, 85]. Although the perceived harm of cigar products among youth seems to increase with age, there remains a subset of individuals, especially those experiencing homelessness, who are less likely to perceive LCCs as harmful as cigarettes.

#### Perceptions of cigar smoking related to specific health outcomes

Three studies measured the perceived harmfulness of cigar products related to specific health outcomes. Participants believed flavored LCCs to be less harmful [83], less likely to cause cancer [83], and less dangerous than cigarettes in regards to developing cancer or having a heart attack [81]. An online survey among high school students found that youth rated cigars as similarly harmful to cigarettes, perceiving cigar smokers to be at high risk for developing a variety of short and term-long term health effects including, bad cough, oral and lung cancer, and heart attacks [82]. The discrepancies in findings between these three studies [81–83] are likely due to the differences in the target population. Specifically, the first two studies [81, 83] assessed harm perceptions among a sample of adult established LCC users who may be conditioned to cigar smoking, and as a result, express a lower harm perception. The third study [82] assessed harm perceptions among an adolescent sample of mostly tobacco naïve individuals, who may be more receptive to the ongoing public health campaigns regarding the negative health impact of tobacco use, had higher harm perceptions [88].

### Appeal and harm perceptions associated with flavored cigars

The abundance of flavored cigar products contributes to their appeal and initiation of use. High-intensity sweeteners are often applied to the tops of flavored and unflavored cigarillos and at levels that are similar to those used in sugar-free candy and gum [89]. Sweeteners placed on the mouth tip of the cigar allows for a more pleasant smoke by reducing the inherent harshness and may contribute to the appeal of cigarillos. Consistent with this, among high school students who reported using cigarillos, appealing flavors was the primary reason cited for trying a cigarillo, followed by curiosity [90].

In focus group interviews among 90 college students who used LCCs and cigarettes [40], nearly all participants reported that flavorings made LCCs more pleasant and improved their moods. Over 60% of participants reported initiation of LCCs with a flavored product, and most participants reported the use of flavored LCCs in the past 30 days [40]. Using the same qualitative design and sample, the display of flavors on the LCC packaging was found to contribute to beliefs that LCCs are safer and more “natural” [83].

Data from a U.S. national multi-ethnic sample of 964 adults who smoke cigarettes revealed that individuals who had positive perceptions of flavor additives were more likely to smoke flavored LCCs [86]. A survey of adults in the U.S. found that Black persons who smoke cigarettes, individuals with lower levels of educational attainment, and individuals who use menthol cigarettes, had greater odds of believing that flavored LCC smoking was less harmful than smoking cigarettes [84]. Using data from the 2015 Tobacco Products and Risk Perception Survey in the U.S., adults who perceived flavored LCCs to be lower risk than cigarettes were more likely to have ever used flavored LCCs compared to those who reported that they did not know the extent of the risk [17]. Thus, the availability of flavors in cigar products contributes to the uptake in LCC use and the misperception that flavored LCCs are less harmful than cigarettes.

### The cardiopulmonary health effects of cigars

Fifteen publications evaluated the relation between short- and long-term cardiopulmonary health measures and outcomes associated with cigar use (Table 4).

**Table 4:** Studies Evaluating the Cardiovascular and Pulmonary Health Effects of Cigar Products

Author	Publication Year	Country	Cohort	Follow-up	Total Participants	Total Current Cigar Users	Cigar User Classification	Age Range, years	Inclusion of Females	Inclusion of Non-White Individuals	Cardiopulmonary Measure/Outcome
Nonnemaker [91]	2014	US	National Adult Tobacco Survey, American Cancer Society’s Cancer Prevention Studies I and II	2009–2010	~ 100,000	23,079	Any cigar product	≥ 35	Y	N	Mortality
Taghizadeh [92]	2016	Netherlands	Vlagtwedde-Vlaardingen	2009	8,465	NA	Pipe/cigar	≥ 30	Y	N	Mortality
Christensen [93]	2018	US	National Longitudinal Mortality Study	2011	357,420	1,139	Grouped users of cigars, cigarillos, little cigars	≥ 35	Y	Y	Mortality
Salloum [103]	2019	US	PATH Survey	2013–2014	1,527	NA	Any cigar product	≥ 18	Y	Y	History of cancer
Sutter [94]	2018	US	Virginia Youth Survey	2013	1,168	236	Any cigar product	High school students	Y	Y	Asthma diagnosis
Blank [58]	2015	US	NA	NA	20	20	Black & Mild cigarillo	18–35	Y	Y	Heart rate, blood pressure
Claus [6]	2018	US	NA	NA	77	77	Any cigar product	22–77	Y	Y	Heart rate, blood pressure, respiratory rate, SpO_2_
Strong [61]	2018	US	PATH Survey	2013–2014	17,952	706	Any cigar product	≥ 18	Y	Y	History of respiratory disease
Stanton [97]	2016	US	US National Survey on Drug Use and Health	2005–2013	335,080	26,827	Any cigar product	≥ 18	Y	Y	Chronic health conditions
Murgia [95]	2019	Italy	Cooperative Health Research In South Tyrol	2011–2017	4,751	860	Any combustible tobacco product	18–93	Y	N	Heart rate variability
Reed [96]	2017	US	Amish Community	2001–2015	3,568	519	Any combustible product (cigars, cigarettes, pipes)	≥ 18	N	N	Heart rate variability, spirometric lung function, ankle-brachial index, aortic diameter, cholesterol, obesity
Gaalema [98]	2018	US	PATH Survey	2013–2015	45,971	2,753	Any cigar product	≥ 18	Y	Y	Self-report of myocardial infarction

NA = Not available/applicable

#### Association of cigar use with cardiopulmonary outcomes

Using the National Adult Tobacco Survey data from 2009 to 2010 and the American Cancer Society’s Cancer Prevention Studies I and II, the mortality relative risks for cancers of the trachea, lung, and bronchus were 5.1 for current cigar smokers and 1.6 for former cigar smokers compared to never smokers [91]. Of note, cigar smokers who also smoked cigarettes were included in the cigar use group. Using a sensitivity analysis, individuals who reported moderate to deep inhalation had mortality risks of 5.9 for current cigar smokers and 1.6 for former cigar smokers compared to never-smokers. Both current and former cigar smokers had mortality relative risks of 1.4 for COPD; however, when the analysis was restricted to those reporting moderate to deep inhalation, the risk of COPD increased to 4.5 for current cigar smokers and 4.2 for former cigar smokers, suggesting that the depth of inhalation contributes to the cigar smoking-associated risks. In a sensitivity analysis excluding dual cigar and cigarette users, both frequent (≥ 15 of the past 30 days) and less frequent (1 day in the past 30) use with moderate to deep inhalation more than doubled the smoking attributable mortality. Even less frequent cigar smoking was associated with an increased risk of mortality [91].

In a sample of White individuals of Dutch descent, the hazard ratios (HR) associated with cigar smoking for cardiopulmonary diseases were examined [92]. Individuals who also smoke cigarettes were not included in the pipe and cigar use group for this study. Males who never smoked tobacco had a lower risk of all-cause mortality, cardiovascular disease, COPD, and cancer (lung/breast/prostate/colorectal)-related mortalities compared to males who smoked pipes/cigars. Males who smoked pipes/cigars throughout their lifetime had a greater risk of lung cancer, all-cause and cancer-related mortality.

Using the National Longitudinal Mortality Study, the HR for lung cancer, respiratory disease, and COPD among former and current exclusive cigar smokers was estimated compared to never tobacco users [93]. In multivariable models, current cigar smokers had higher risks of all-cause and cancer-related mortality compared to never tobacco users. Stratification by non-daily and daily use revealed that daily cigar use was associated with higher mortality risks from tobacco-related cancer, lung cancer, and COPD. Evaluation of the mortality risk associated with cigar smoking due to other specific causes of death, including cardiovascular disease-related mortality, were limited by a small number of events and modest sample sizes.

#### Association of cigar use with respiratory diseases and chronic health conditions

Among latent classes of youth who use tobacco, including a class characterized by high probabilities of cigar use and low probabilities of cigarette/smokeless tobacco use (20.9% of the sample), asthma diagnosis status was similar across all tobacco user classes [94]. In another cross-sectional study, cigar users were more likely to report a history of respiratory disease compared to non-tobacco users, regardless of their cannabis use status [61].

#### Association of cigar use with biomarkers of harm

One clinical study examined heart rate, blood pressure, oxygen saturation, and respiratory rate (breaths per minute) following acute cigar smoking (own-brand cigar product) in current cigar smokers [6]. Heart rate, blood pressure, and respiratory rate increased after cigar smoking, while oxygen saturation decreased. Other than oxygen saturation, none of the outcomes differed by cigar size, cigarette smoking history, or self-reported inhalation behaviors, suggesting that all cigar products have similar effects on these acute cardiopulmonary measures. Secondary cigar smokers (smoked ≥ 100 cigarettes in their lifetime) had smaller decreases in oxygen saturation compared to primary cigar smokers (smoked < 100 cigarettes in their lifetime), which is likely the result of differences in smoking topography. Primary cigar smokers smoked for longer and took shorter duration puffs compared to secondary smokers.

In the Cooperative Health Research in South Tyrol Study, current smoking of combustible tobacco was associated with lower heart rate variability, a predictor of cardiac events, indicative of increased sympathetic activation [95]. A dose-response effect was noted with increasing grams of daily tobacco consumed inversely associated with lower heart rate variability [95]. Another clinical study examined the cardiovascular effects of acute Black & Mild cigar smoking under unmodified and “hyped” conditions as compared to sham smoking [58]. Puff topography measures and plasma nicotine levels were similar for the modified and unmodified cigar conditions. Smoking either a hyped or unmodified cigarillo resulted in similar increases in heart rate, suggesting that the removal of the tobacco liner was not associated with reduced cardiovascular harm.

The Amish population has a high prevalence of cigar use among men. In a cross-sectional study of participants in an Amish community, 34% of men reported ever smoking, among whom, 64% reported little cigar use [96]. Combustible tobacco smoking was associated with higher body mass index, weight, waist-hip ratio, and triglycerides, and lower high density lipoproteins (HDL). The FEV_1_/FVC ratio, the percentage of the FVC expired in one second, was lower in smokers compared to never smokers suggestive of obstructive pulmonary disease. Further, smokers had lower ankle-brachial indices and greater aortic root sizes compared to males who never smoked, indicative of vascular remodeling [96]. Although little cigar use was high in the sample, all combustible products were grouped together, limiting the ability to delineate the effects of little cigars versus cigarettes.

#### Prevalence of cigar use among individuals with chronic health conditions

In a cross-sectional analysis using multiple waves of the PATH Study (2005–2013), cigar smoking was higher among individuals reporting chronic health conditions compared to those without chronic health conditions, and cigar smoking was more prevalent among those with asthma [97]. In another PATH Study (2013–2015), adults who reported a myocardial infarction were more likely to report smoking combustible tobacco products, including cigars [98]. Patients who reported a chronic health condition or cardiovascular disease risk factors were more likely to believe that tobacco use caused or worsened their health condition compared to adults without a chronic condition [98]. Despite the recognition of the harm of tobacco use, patients who had a recent myocardial infarction did not report changes in their tobacco use. Although patients with chronic conditions were more likely to be counseled to quit tobacco use compared to patients who do not use tobacco, the patients that used non-cigarette products were less likely to be counseled on quitting tobacco use [99].

#### In vitro cigar exposures induce toxicity in pulmonary and cardiovascular cell types

We identified two studies that evaluated the effects of in vitro exposure to little cigar smoke and flavoring additives on cardiovascular and pulmonary cell types. In bronchial epithelial cells, cigar smoke exposure resulted in greater cytotoxicity, differential gene expression profiles, and greater proinflammatory cytokine secretion compared to cigarette smoke exposure [98]. In vitro exposure of endothelial cells to flavoring additives commonly used in tobacco products induced endothelial dysfunction characterized by a loss of nitric oxide production, with several of the additives upregulating proinflammatory interleukin 6, even at the lowest dilutions tested [100].

## Conclusion & future directions

Cigar use continues to rise, especially among groups that have been economically and socially marginalized, and those who have been historically targeted by tobacco company marketing (e.g., younger consumers and those who identify as Hispanic and/or Black), as does the misperception that cigars confer less risk than cigarettes [3, 16, 25, 27–30, 61–66, 71, 74, 77]. Flavored cigars are widely available and appeal to users, especially youth and co-substance users, by masking the harsh taste of tobacco [80, 83, 88]. The availability of flavored cigars contributes to the misperception of reduced harm of LCC [17, 81, 83]. The emergence of LCCs has driven an increased prevalence of cigar use and more regular use patterns (daily and every few days use compared to infrequent use). We summarize the key findings in Table 5.

**Table 5:** Key Findings

**Cigar Use Patterns**
· Higher prevalence of cigar smoking among young adults, males, those reporting a lower income, and Non-Hispanic Blacks in the US
· Poly-tobacco and substance use is common among those who report using cigars
· Flavors in cigars contribute to youth and young adult use
**Smoking Topography, Biomarkers of Exposure, & Nicotine**
· Smoking topography differs by cigar type with studies suggesting self-titration to achieve similar nicotine exposure
· Despite differences in smoking topography by cigar type, biomakers of exposure are similar for cigars and cigarettes with a few exceptions
· Nicotine levels in little cigars is similar/higher than those in cigarettes
· Large cigar and cigarillo wrappers contain measurable nicotine levels, which has implications for “hyping” modifications
**Perceptions of Harm of Cigar Smoking**
· Most adults believe cigar smoking to be as harmful as cigarette smoking
· Youth and individuals from groups that have been socially marginalized perceive cigars as less harmful than cigarettes
· Flavors contribute to misperceptions that cigars are safer than cigarettes
**Cardiovascular & Pulmonary Health Effects of Cigars**
· Cigar smoking is associated with ↑ mortality risk of cancers of trachea, lung, and bronchus, all-cause cancer, and COPD
· ↑ all-cause mortality associated with cigar use is higher for those reporting deeper inhalation and more frequent use (≥ 15 days of the past 30 or daily use)
· Greater likelihood of reporting respiratory disease, chronic health conditions, and myocardial infarction among individuals who use cigars
· ↑ heart rate, blood pressure, and respiratory rate and ↓ oxygen saturation post-cigar use
· Higher BMI, weight, waist-hip ratio, triglycerides, aortic root size and lower FEV1/FVC ratio, HDL, ankle-brachial indices among male smokers of combustible tobacco (grouped cigars and cigarettes) compared to males who never smoked tobacco
· Individuals who had a recent myocardial infarction were less likely to be counseled to quit tobacco if they used non-cigarette products, including cigars
· Flavoring additives used in cigar products induce bronchial epithelial cell and endothelial cell toxicity

Although historically, cigar smoke was held in the mouth rather than inhaled, contemporary studies indicate that cigar use has changed with most individuals inhaling the cigar smoke [51–53]. The smoking topography for little cigars is similar to that of cigarettes, which is likely due to the close similarity in size, shape, and composition of little cigars to cigarettes [51, 59]. While cigarillos are large in size, like premium cigars, the presence of flavorings and sweeteners is common, which makes the smoke of these products easier to inhale compared to premium cigars [80, 83, 88]. Additionally, individuals who use two or more tobacco products, including cigars, are more likely to inhale cigar smoke in a manner more similar to cigarette smoking compared to individuals who only smoke cigars [52, 53, 101]. The product characteristics of the newer cigar products (little cigars, cigarillos) likely contribute to the inhalation and differences in smoking topography of these products compared to premium cigars.

The mainstream smoke generated by LCCs contains similar levels of toxicants and carcinogens as cigarettes and LCC use is associated with similar levels of biomarkers of exposure as cigarettes, with a few exceptions, including CO [20, 21, 102]. Therefore, along with the data indicating inhalation of cigar smoke, it is reasonable to conclude that cigar products likely convey similar health risks as cigarettes. Emerging studies evaluating the cardiovascular and pulmonary health effects associated with LCCs are limited but suggest similar health risks are conferred by LCC smoking as cigarette smoking. Specifically, cigar use is associated with alterations in vascular function, heart rate, and spirometry, suggestive of cardiovascular and pulmonary toxicity [6, 58, 95, 96]. Importantly, cigar smoking is associated with adverse health outcomes, most notably all-cause and cancer-related mortality, especially among individuals reporting more frequent and deeper inhalation [91–93].

We identified several limitations in the studies evaluated. Historically, most studies evaluating the cardiopulmonary health effects of cigar use have been largely restricted to White men, which is not reflective of the contemporary users of cigars. Many current users of cigar products include youth, young adults, and individuals who identify as Black and/or Hispanic. It is essential to focus future studies on these groups of individuals to fully understand and appreciate the potential health toxicity, use patterns, and use behaviors surrounding LCC products. Additionally, cigar products are typically grouped into a single category or with other tobacco products, creating challenges in teasing apart the health risks conveyed by specific types of cigars. Grouping all cigar products together fails to account for the product-specific differences in cigar smoking frequency, intensity, or topography. Most studies identified in this systematic review were conducted within the US and hence, the findings may not be applicable to all countries where the cigar use patterns and product characteristics may differ, which requires additional investigation. Further, many of the studies demonstrating elevated cardiovascular and pulmonary disease risks associated with cigar smoking are based upon the evaluation of outcomes in individuals who report using both cigars and cigarettes, which confounds the ability to attribute the health risks solely to cigar use. Future studies evaluating the cardiopulmonary health effects of the different cigar products are needed, especially among diverse population samples that are more reflective of contemporary use patterns.

Evaluation of the cardiopulmonary health effects of cigars is challenging due to product diversity. Consequently, one specific brand or cigar product is often selected for interventional research study visits, which may not be reflective of the participant’s desired product or elicit the native use behavior of the participant. The prevalence of exclusive cigar use is low and therefore large population studies of the cardiopulmonary health effects of individual cigar product classes may be difficult. A high prevalence of dual, poly, and co-substance use among users of cigar products makes it difficult to attribute the health effects to individual products. Additionally, co-use of LCCs with cannabis and alcohol is prevalent among cigar users, especially young adults, and Black individuals. Taken together, the limitations in the studies evaluated highlight the need for a more detailed inquiry into the related health risks relevant to each individual cigar product category.

The perceived harm and addictiveness of cigars varies based upon the populations sampled. Most adults perceive cigars to be equally harmful and addictive as cigarettes. In contrast, youth and young adults, individuals experiencing homelessness, Black individuals, and SGM individuals, perceive cigar products to be less harmful and less addictive than cigarettes. The reduced perceived harm of cigars among high-risk groups underscores the urgency for increased education and prevention programs. Intervention efforts surrounding non-cigarette tobacco products must be implemented to provide the best educational resources and tools needed to help individuals with smoking cessation to improve health outcomes.

With a toxicant composition comparable to cigarettes, cigars should be regulated similarly to cigarettes to protect public health and prevent widening tobacco-related health disparities. In April 2021, the FDA announced an initiative to ban characterizing flavors, including menthol, in cigars. Studies have found that the most popular flavor categories for cigars are fruit, alcohol, and sweet flavors, which are not currently available for cigarettes [34, 35, 40, 43]. LCC packaging with colorful wrappers and the wide array of available flavors has contributed greatly to their appeal, increased LCC initiation, and susceptibility of use among youth and young adults [40, 46]. Additionally, flavorings and sweeteners make LCCs more pleasant to use by masking the harsh flavor of the tobacco, contributing to a perception of reduced harm of LCCs compared to cigarettes [83]. Prohibition of characterizing flavorings and sweeteners in cigars is an indispensable target for intervention. The proposed FDA initiative introduces the opportunity to reduce youth initiation, increase cessation efforts, and address health disparities experienced by communities of color, those who are economically disadvantaged, and SGM individuals.

## Data Availability

The datasets used and/or analyzed are included in this published article.

## List of abbreviations

CO: Carbon monoxide
COPD: Chronic obstructive pulmonary disease
FDA: Food and Drug Administration
FEV: Forced expiratory volume
FVC: Forced vital capacity
HDL: High density lipoprotein
HR: Hazard ratio
LCC: Little cigar and cigarillo
NNAL: 4-(methylnitrosamino)-1-(3-pyridyl)-1-butanol
PATH: Population Assessment of Tobacco and Health
SGM: Sexual and Gender Minority
U.S.: United States
